# A Review on Chromatography–Mass Spectrometry Applications on Anthocyanin and Ellagitannin Metabolites of Blackberries and Raspberries

**DOI:** 10.3390/foods10092150

**Published:** 2021-09-11

**Authors:** Dilip K. Rai, Katerina Tzima

**Affiliations:** Department of Food BioSciences, Teagasc Food Research Centre, Ashtown, D15 DN3K Dublin, Ireland; dilip.rai@teagasc.ie

**Keywords:** anthocyanins, ellagitannins, blackberries, raspberries, metabolites, liquid chromatography, mass spectrometry

## Abstract

Berries have been widely assessed for their beneficial health effects, predominately due to their high (poly)phenol content of anthocyanins and ellagitannins. After ellagitannins and ellagic acid are metabolized by the gut microbiome, a class of compounds known as urolithins are produced, which exert potential advantageous health effects. Anthocyanins, on the other hand, undergo a complex metabolic pathway after their interaction with microbial and endogenous enzymes, forming a broad range of metabolites and catabolic products. In most cases, in vitro models and cell lines are used to generate metabolites, whereas their assessment in vivo is currently limited. Thus far, several analytical methods have been developed for the qualitative and quantitative analysis of phenolic metabolites in berries, including liquid chromatography, mass spectrometry, and other hyphenated techniques, and have been undoubtedly valuable tools for the detailed metabolite characterization and profiling. In this review, a compilation of studies providing information on the qualitative and quantitative analysis of (poly)phenol metabolites in blackberries and raspberries after the utilization of in vitro and in vivo methods is presented. The different analytical techniques employed are assessed, focusing on the fate of the produced metabolic compounds in order to provide evidence on their characteristics, formation, and beneficial effects.

## 1. Introduction

The biological activity of dietary (poly)phenols depends on their bioavailability and catabolism [1]. (Poly)phenol metabolism is a process of high complexity [2,3], where the (poly)phenols interact with various biomolecules, such as digestive enzymes and cellular transporters that are present in the gastrointestinal (GI) lumen, enterocytes, and liver, prior to entering the systematic circulation [4]. (Poly)phenols in berries are constituted largely of anthocyanins and ellagitannins [5,6]. It is speculated that (poly)phenol bioaccessibility from berries is greater compared to other fruits, owing to their higher abundance but lower content of other macromolecules, including indigestible carbohydrates and proteins [7]. The genus *Rubus* L. constitutes a highly diverse plant genus [8], with species such as *Rubus fruticosus* (blackberry) and *Rubus idaeus* (raspberry) having a high polyphenolic content [9,10,11], and with compounds including anthocyanins, ellagitannins, and ellagic acid conjugates to be amongst the most prevalent (poly)phenols [9,11,12,13]. Anthocyanins in blackberries and raspberries are responsible for their characteristic pigmentation, with cyanidin 3-O-glucoside (C3G) being predominant in blackberries, whilst cyanidin 3-sophoroside in raspberries [2,9,14,15,16]. Currently, blackberries comprise one of the leading berry plants worldwide [17]. Similarly, the global demand for raspberries has significantly increased either for direct consumption or as a constituent in food industrial applications [18]. The high popularity of these species has also been reflected on the continuous scientific interest in them, which can be highlighted by various recent studies focusing exclusively on the effects of those two berries on different contexts [19,20,21,22,23]. However, the potency of other species of the *Rubus* genus such as the growing in popularity Nordic *Rubus chamaemorus* (cloudberry) [24] should not be dismissed.

The consumption of blackberries has been associated with neuroprotective, hypoglycemic, hypolipidemic, antioxidant, anti-inflammatory, anticancer, and cardioprotective effects [14,25,26,27,28,29], whereas similar beneficial effects have been also ascribed to raspberries [30,31,32,33,34]. Despite their proven health benefits [35,36], individual anthocyanins are usually identified in plasma in a concentration of ~1% of the total consumed amounts due to low intestinal absorption [3]. However, other parameters may affect their bioavailability, including the increased rates of cellular uptake, metabolism, and excretion [3]. The limited absorption and gut accumulation have led to the assumption that their metabolites are the ones responsible for the beneficial effects [35]. Nonetheless, the related mechanisms, which are associated with the bioactivity of anthocyanins and phenolic compounds entail various pathways [9]. There is currently a limited knowledge in anthocyanin transportation across the gastric barrier or blood–brain barrier (BBB) [37]. Hence, more studies are required to underline the transport of these compounds across biological membranes and examine the gastric absorption of anthocyanin-rich foods [37]. On the other hand, ellagitannins are highly underestimated nutritionally as a result of their capacity to precipitate protein molecules, even though in a substantially lower extent to that of proanthocyanidins and gallotannins [38]. Due to their structural complexity, ellagitannins are also not highly bioavailable [39]. Despite not being detected in plasma or other biological fluids, the ellagitannins have shown potential beneficial effects throughout the gastrointestinal tract (GIT) [39]. Therefore, there is an increasing awareness that colonic catabolites, such as phenolic acids and urolithins, may have a significant contribution in the beneficial effects of a fruit-rich diets [40]. However, as phenolic compounds are subjected to an extensive metabolism in the human body, their bioavailability is low in comparison to the produced metabolites. Hence, taking into account the biological activity of only the parent (poly)phenols and not their metabolites, is a major issue, particularly in in vitro studies [41].

Versatile, sensitive, and selective analytical techniques such as liquid chromatography (LC) coupled to tandem mass spectrometry (MS/MS) have enabled the qualitative and quantitative analyses of the (poly)phenolic compounds present in different plant matrices, as well as their metabolic products [42,43,44]. The emergence of high-resolution instrumentation including orthogonal quadrupole time-of-flight (Q-TOF) MS over the last two decades has enabled high sensitivity, mass accuracy, and dynamic range in LC–MS/MS analysis [45]. LC coupled to fluorescence or electrochemical detection systems has also shown satisfactory sensitivity in contrast to ultraviolet/visible (UV–VIS) detectors. Nonetheless, MS is a superior detector to all in conjunction with LC, in the quantification of analytes [30]. Targeted LC–MS/MS methods have been employed to identify different metabolites on the basis of a theoretical extrapolation or predicted metabolic pathway of potential metabolites and their conjugates [46]. Untargeted metabolomics profiling of metabolites on the basis of their structural similarity to a parent compound, or when their MS/MS spectra match those present in the compound library has also been reported [45,47]. At the same time, the use of both positive and negative ionization modes [7] in MS enables increased coverage of identification of metabolites [45]. However, the lack of metabolite standards creates restrictions in the assessment of (poly)phenol metabolism, particularly when absolute quantitative determination is required [48,49]. Even though LC–MS has enabled the detection, qualitative and quantitative analysis of (poly)phenols and their metabolic products, high-through-output analytical platforms have been recommended by the European Cooperation in Science and Technology Commission led COST Action FA-1403 POSITIVe [44,45]. However, the application of different methodologies in the existing studies in conjunction to the high number of metabolites that are present in them have led in difficulties associated with the collection and organization of all the metabolites and metabolic pathways [44].

Several researchers have focused on the assessment of metabolites in *Rubus* genus, with the vast majority of the studies comprising those entailing on the metabolic pathways after in vitro and in vivo digestion and fermentation of blackberries and raspberries. Previous work has reviewed the chemical properties and health effects of anthocyanins on cardiovascular and neurodegenerative diseases focusing among others in a number of general notions regarding their chromatographic analysis and bioavailability [50]. Other researchers have reviewed the general structure, presence, and biological aspects of ellagitannins from various sources [51], whereas some have focused on their general metabolism, functions, and health effects in conjunction to those of ellagic acid and the produced metabolites [52]. Nevertheless, to the best of our knowledge, no literature review encompassing the formed metabolites of their main (poly)phenols in conjunction to the analytical techniques utilized has been conducted thus far in blackberries and raspberries. Therefore, this review aims to summarize the up-to-date applications of advanced analytical techniques on (poly)phenol metabolites from blackberries and raspberries after enzymatic and microbial action, focusing on anthocyanins and ellagitannins, in parallel to the absorption and metabolism of (poly)phenols.

## 2. Absorption and Metabolism of (Poly)Phenols

It has been generally observed that the absorbability and circulatory concentration of (poly)phenols are substantially low in comparison to their corresponding metabolites. Therefore, these serum-bioavailable metabolites tend to be much more potent in overcoming cellular barriers and reaching target tissues, such as the brain [53]. The epithelial tissues of luminal organs, such as the GIT, constitute a controlled and selective permeability barrier among the luminal factors, comprising the nutrients, secretions, and essential compounds produced in the GI lumen, as well as among the intrinsic tissue sections [54]. Besides the occurrence of (poly)phenol absorption in both the stomach and small intestine by diffusion or transport [2,3], the small intestine plays the most crucial role in their bioavailability [55,56]. The bioavailability of (poly)phenols is highly associated with their structural characteristics, the food source in which they are present, and also the co-existence of anti-nutritional factors with the capacity that hinder their release, and intestinal absorbability [4]. For instance, after ingestion, the presence of glycoside groups in (poly)phenol-glycosides require prior hydrolysis in small intestine, whereby the cleaved sugar moieties enter systematic circulation [55,57]. This process can take place through two potential mechanisms: (1) the action of lactase-phlorizin hydrolase or (2) cytosolic β-glucosidase, which are present in the enterocyte membrane and have the capacity to stimulate the hydrolysis of glycosylated forms producing the aglycones that can enter the epithelial cells by passive diffusion (PD) [55,57]. After absorption, (poly)phenols are bio transformed throughout the phase II enzymatic conjugation either in the small intestine itself or in the liver [36]. Since the majority of dietary (poly)phenols are not efficiently absorbed in the small intestine, as they exhibit a resistance in the action of lactase-phlorizin hydrolase or cytosolic β-glucosidase, several dietary phenolic compounds can reach the colon in varying concentrations, both in their intact form or after their secretion in bile salts, stemming from the enterohepatic cycle [58]. The colon, with its microbiome, constitutes an additional active site, where radical changes of polyphenol structures are stimulated and are relatively different from those produced by intestinal and hepatic enzymes [1,56]. Hence, except for hydrolysis of complex polyphenol glycosides [58] or other forms such as glucuronides, sulfates, amides, esters, and lactones [59], colonic microbiome can also convert their aglycone structures into new molecules [1,58], which can be smaller and more easily absorbable (e.g., hydroxyphenylacetic acids) than the aglycones [1,57]. These small metabolites can further lead to an elevated or synergistic bioactivity and therefore result in positive health effects [7].

**Figure 1:**
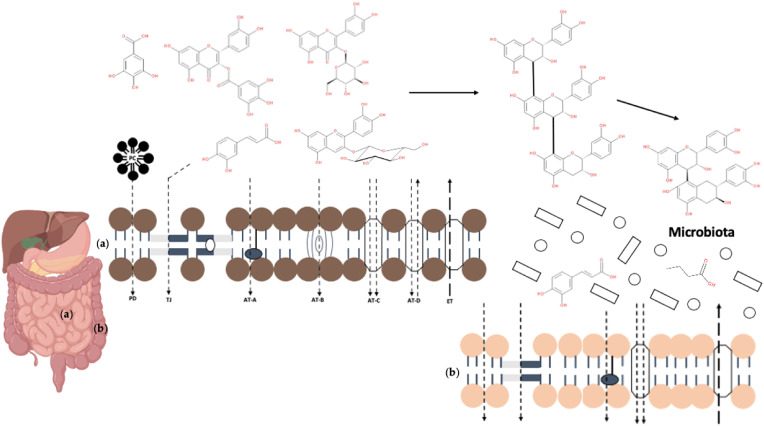
The hypothetical phenolic transport mechanisms as adapted with light modifications by the illustration in the work of Domínguez-Avila et al. [4] in (**a**) small intestine and (**b**) large intestine, where PD corresponds to passive diffusion; TJ to paracellular in tight junctions; ET to efflux transport; and AT-A, AT-B, AT-C, and AT-D to single-solute, translocase-type, co-solute symporter, and antiporter active transporters, respectively. Reproduced from Ref. [4] with permission from the Royal Society of Chemistry.

An in-depth demonstration of the different transcellular mechanisms in the small and large intestines has been reviewed by Domínguez-Avila et al. [4]. According to the authors, after their release, both hydrophilic and lipophilic phenolic compounds are transported by the various transcellular mechanisms including PD, carrier-mediated active transport (AT), and paracellular transport in tight junctions (TJ), as illustrated in Figure 1, which describes “the hypothetical phenolic transport mechanisms” based on their study. However, there is still limited knowledge with respect to different absorption pathways, such as for those involving active, passive, or paracellular routes, but also for the influx and efflux transporters (ETs) in the epithelium [47]. For instance, according to the same authors, a significantly higher absorbability of various (poly)phenols has been observed in the presence of others, as they have the capacity to block the gut ETs that generally decrease the intracellular concentration of such “xenobiotics”. As it has been also suggested, several phytochemicals are considered as “xenobiotics” and are expelled from the cell, typically by increasing their polarity and via ETs. Currently, limited information is available for the specificity of these transporters, and even less on the capacity of phytochemicals to block them and lead to higher bioaccessibility [4].

Previous work has demonstrated that differences in the endothelial transport may be associated with the degree of the occurring chemical changes, whereas the combination of methylation and sulfation has exhibited improved transport of the bioavailable (poly)phenol metabolites [60]. Conjugation reactions, and mainly glucuronidation, sulfation, and methylation, are known to take part the metabolism of phenolic compounds in the human body, whereas they mainly result in stabilisation and high water solubility, leading to changes in their distribution and excretion [61]. Molecules with low molecular weight such as phenolic acids and catechins have generally exhibited a higher degree of absorbability by the epithelium, whereas those of high molecular weight and high degree of polymerization, including proanthocyanidins and ellagitannins, are not absorbed [62]. Anthocyanins and other phenolic compounds in glycosylated forms using AT as their PD is less feasible [4]. At the same time, TJs play a significant role in the intestinal barrier function [63] as they comprise the apical most intercellular junction in both epithelial and endothelial cells [54], which regulate the permeability of the intestinal barrier [64,65]. TJs are comprised of transmembrane proteins that are connected to a cytoplasmic plaque, with the latter being formed by a complex of scaffold and signal transducing adaptor proteins, signalling components and actin-binding cytoskeleton linkers [65]. It has been suggested that uncharged and lipophilic phenolic compounds, in parallel to other nanoparticles, can easily pass through the narrow TJ via specific proteins known as claudins [4]. The high electrical resistance that these proteins offer to TJ is the principal reason for the use of this transport system for low molecular weight and charged phenolics (e.g., gallic acid) [4]. The similarity in the CaCo-2 epithelia cell-line permeability of epigallocatechin-3-gallate (0.22 nmol/min × mg protein) with gallic acid (0.16 nmol/min × mg protein) suggests that the former can be also absorbed intact through the TJ transport system [4].

Since 2007, it has been recommended that ETs such as breast cancer resistance- and multidrug resistance-associated proteins, which enable the removal of hydrophilic phase II conjugates, are crucial for estimating the bioavailability of dietary (poly)phenols [66]. ETs enable the transfer of metabolites of xenobiotics out of the cells [67]. However, these transporters also protect sensitive tissues (i.e., brain) from the entry of xenobiotics as they are present in the BBB [67]. Higher efflux for flavanols has been shown to result in limited bioavailability, whereas a higher potential of hydroxycinnamate absorption has been ascribed to their greater permeability through the intestinal epithelial cell monolayers [68].

For instance, the increased efflux has been speculated to be the reason for the very low efficiency of free quercetin transport at the apical membrane, which was approximately 8% after 18 h of incubation. Nonetheless, this effect may be also attributable to a low transport rate through the basolateral membrane, as well as its high metabolism or degradation rate [69]. Several in vitro studies have assessed the bioactivity of different (poly)phenols, their characteristics, and their health effects, while the assessment of metabolic and microbial interactions in vitro and constitutes a continuously evolving research area [70,71]. However, in in vivo studies, some of the data obtained *in vitro* cannot be entirely reproduced, whereas a substantial variability is additionally observed among different subjects. Therefore, bioavailability and bioefficacy studies on phenolic compounds still require extensive investigation [71]. Various techniques including LC, ultra-high performance liquid chromatography (UPLC), and gas chromatography (GC), commonly coupled with MS or sometimes aided by nuclear magnetic resonance (NMR) spectroscopy, are used for the structural characterization of the metabolites in in vitro and in vivo studies.

## 3. Anthocyanins and Their Metabolites in Blackberries and Raspberries

The health-beneficial properties after consumption of blackberries have been associated with their high anthocyanin contents [72]. Anthocyanins constitute one of the most abundant flavonoid classes in plants, which are responsible for the pigmentation of plant matrices [73,74]. Currently, more than 500 different types of anthocyanins have been reported in the literature, and they have been identified in more than 27 and 72 plant families and genera, respectively [74]. The classification of anthocyanins is mainly based either on differences in the substitution groups of their B-ring or the number of conjugated sugar molecules, and they can be also classified on the basis of the presence or absence of an acyl group on their molecule [73]. Chemical aspects can affect the in vivo stability of components derived from anthocyanins, whereas they can additionally have an impact on their ability to act as substrates for xenobiotic conjugation and transport, but also their connection with the biomatrix [75]. Anthocyanins have been reported to be rapidly rearranged and modified when undergoing digestion and fermentation [76]. It has also been suggested that the sensitivity of anthocyanins to the pancreatic bile digestion is highly dependent on their hydroxylation patterns, but predominately on the type of their sugar moieties [77]. With respect to absorbability, proanthocyanidins have shown the highest degree amongst flavonoids, while the anthocyanins are considered less absorbable in the small intestine; the only exception is the C3G, which has been reported to be similar to other flavonoids [78].

**Figure 2:**
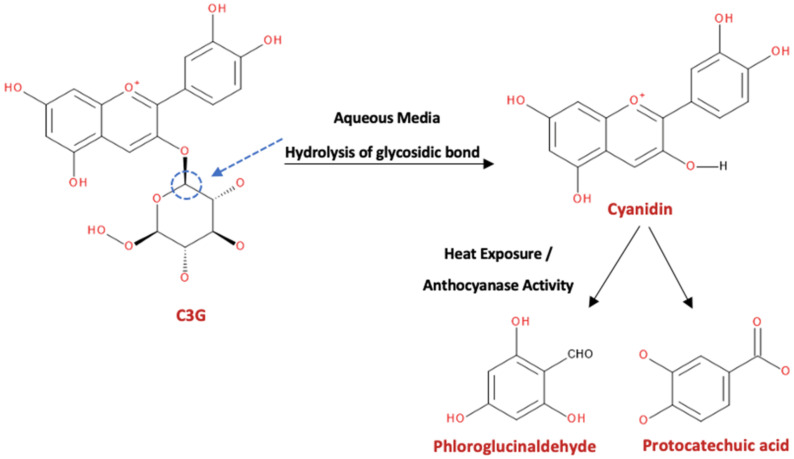
Degradation pathway of C3G according to Dai et al. [8].

According to Dai et al. [9], anthocyanins are degraded through hydrolysis, oxidation, and condensation with other phenolic compounds and are highly influenced by pH and temperature. For instance, the glycosidic bond of the C3G is initially hydrolyzed in aqueous media prior to the opening of its pyrylium ring under heat exposure or anthocyanase activity (Figure 2). Its further degradation results in the formation of protocatechuic acid and phloroglucinaldehyde. In addition, C3G can be co-oxidized with other phenolic compounds, including chlorogenic acid, through the action of polyphenol oxidase, forming o-quinones [9]. The rapid deglycosylation phenomena in anthocyanin molecules in vivo and ex vivo results in the release of anthocyanidins (aglycone forms), which have a lower polarity, and can be absorbed by PD or paracellular diffusion [7]. Nonetheless, after deglycosylation, anthocyanins can also spontaneously break down to produce protocatechuic acid at a physiological pH; hence, colonic microflora are not always essential for the anthocyanin transformation [79]. The advantageous health effects of anthocyanins are related to either their direct effects or indirect effects after absorption of intact compounds and their metabolites [37].

The indirect effects are facilitated by non-absorbed units that potentially stimulate modifications on gut microbiota and therefore on metabolism, or act at the membrane border and stimulate signal transduction pathways [37]. Although there is low absorption and accumulation of anthocyanins in the gut, it is suggested that their gut metabolites are potentially those that exert the beneficial effects [72,80]. Thus, different metabolites may be formed after microbial biotransformation of berry anthocyanins on the basis of the composition of the gut microbiome and, irrespective of specific phenolic acids and flavonoids, might additionally act as prebiotics [78].

**Figure 3:**
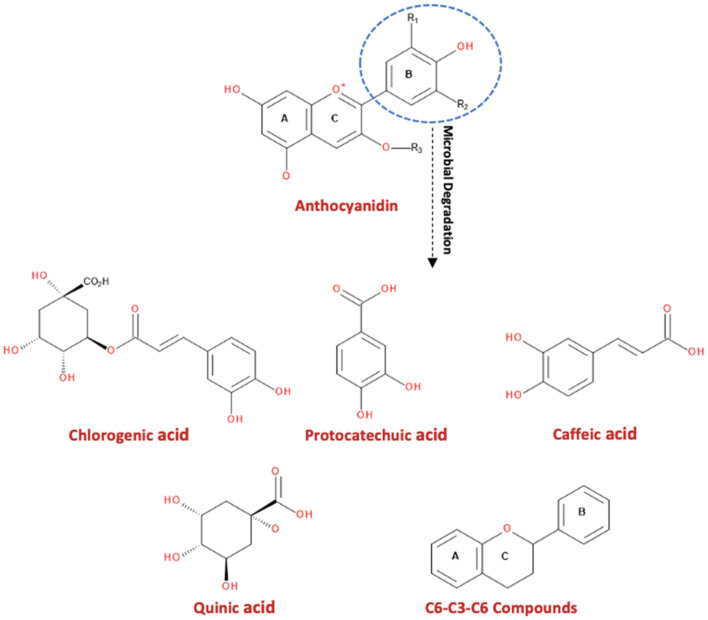
Microbial degraded metabolites of anthocyanidins according to the study of Olivas-Aguirre et al. [7].

Following microbial degradation that acts predominantly on B-ring, anthocyanidins can produce phenolic acids such as protocatechuic, chlorogenic, and caffeic acids, as well as polyols such as quinic acid, and C6-C3-C6-derived intermediates (Figure 3) [7].

**Figure 4:**
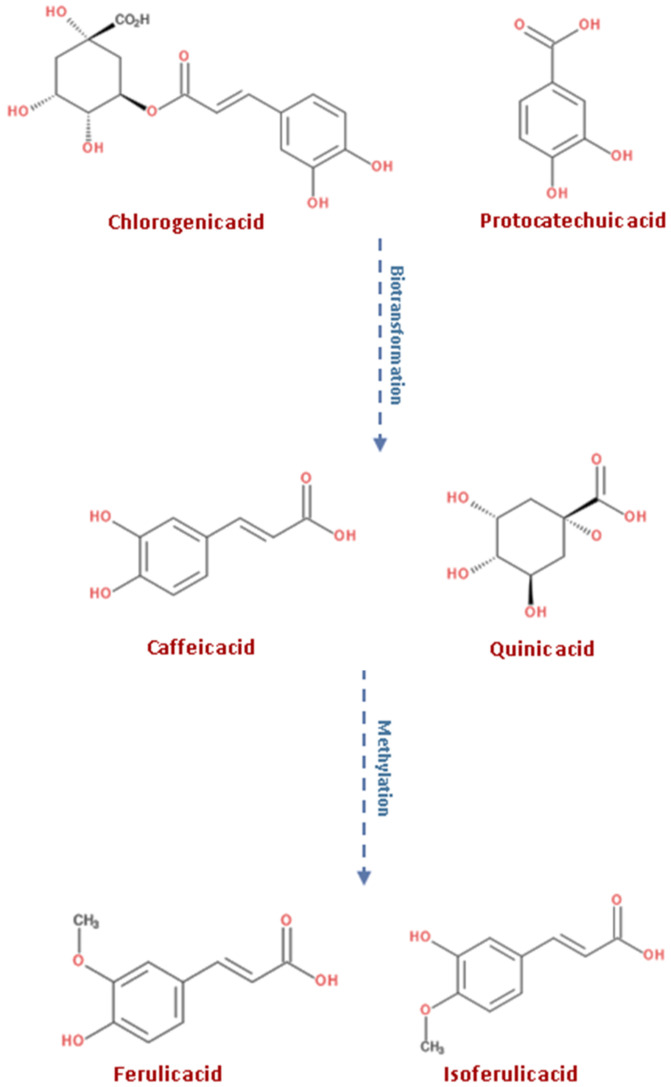
Biotransformation of the main phenolic acids in fruit, namely, chlorogenic and protocatechuic acids, according to the study of Gonthier et al. [81].

On the basis of another study, the biotransformation of chlorogenic and protocatechuic acids, which are two major phenolic acids in fruits, lead in the formation of quinic and caffeic acid, wherein subsequent methylation can produce ferulic and isoferulic acids (Figure 4) [81]. Finally, additional phenolic acids including hippuric, vanillic, and benzoic acid acids can be produced in the colon after the biotransformation of protocatechuic acid [82]. However, as it has been suggested, there is no data supporting the bioactivity of any particular ANT metabolites in vivo [75], whereas many of their positive effects as shown in vitro, have low in vivo relevance [83]. For instance, even if anthocyanins are known for their antioxidant and anti-inflammatory potentials in vitro, their metabolites have shown lower bioactivity after intestinal digestion. This is mainly attributable on the high susceptibility of these molecules to digestion, absorption, and metabolism, and the subsequent formation of metabolites in vivo with different characteristics. On the contrary, other (poly)phenol metabolites exert higher potentials in comparison to their precursor molecules [83]. On the basis of the available literature, various models have been used to assess the produced anthocyanin metabolites of blackberries [1,2,7,35,36,72,84] and raspberries [32,40,85,86,87] in the GIT. Hence, some of the main models will be thoroughly analysed in the following sections.

### 3.1. In Vitro/Ex Vivo Digestion and Fermentation Models

Over recent years, research studies, combining in vitro digestion and absorption with targeted cell assessment, have been used to elucidate the metabolism and bioactivity after consumption of berries, including blackberries [88]. (Poly)phenol metabolites are not only absorbed in the blood circulation, but they can also get attached to the surface of the GIT or get subjected to further colonic fermentation [88]. Hence, numerous dynamic in vitro digestion and fermentation models have been extensively utilized to evaluate the catabolic pathways and bioaccessibility of bioactive constituents, such as (poly)phenolic compounds [89]. In vitro models comprise effective, rapid, and safe methods to mimic GIT processes, being deprived of the restrictions of the in vivo models [90]. These models can deliver essential information with respect to the metabolic pathways of different constituents in the GIT and comprise useful tools in the initial research stages by enabling the provision of direct information on (poly)phenol modifications and metabolites, deprived from the interference of the human physiology [44,89]. Meanwhile, they may also enable the assessment of the potential probiotic effect of these compounds on bacterial cultures [44]. During the last years, human gut models have been also developed and have allowed a thorough insight on the intestinal journey of several compounds. Nonetheless, these models do not mimic the entire GIT, including the effect of the initial stages of digestion, such as the mastication process [44]. In parallel, these models do not have the capacity to evaluate the phenolic compounds that are metabolized by the colonic microflora, and their potential absorption by the large intestine [91]. Hence, the in vitro fermentation models are characterized by limitations as they do not provide the exact human in vivo conditions defined by high density and diversity of the human colonic microbiome [89,92].

Dall’Asta et al. [1] evaluated the in vitro microbial fermentation of a number of high in polyphenols sources, including blackberries, to detect their main metabolites after employing HPLC and electrospray ionisation (ESI)^−^–MS/MS analysis (Table 1) [1]. HPLC coupled with MS/MS has become essential in qualitative and quantitative analysis of polyphenols and their metabolites in different matrices [44,45] due to the high separation resolution of LC and the superior qualitative capacity of MS [93]. The analysis by the previously mentioned authors [1] showed that the blackberry samples contained several (poly)phenolic compounds including pelargonidin hexoside (*m/z* 433) cyanidin hexoside (*m/z* 449), cyanidin malonyl hexoside (*m/z* 535), cyanidin dioxaloylhexoside (*m/z* 593), cyanidin rutinoside (*m/z* 595), coumaroylhexoside (*m/z* 325), caffeoylhexoside (*m/z* 341), quercetin hexoside (*m/z* 463), quercetin glucuronide (*m/z* 477), isorhamnetin glucuronide (*m/z* 491), and salicylic acid (*m/z* 137). However, the only present metabolite detected after 5 and 24 h of in vitro colonic fermentation was protocatechuic acid (*m/z* 153). The authors identified a lack of several digestive phases prior to colonic fermentation was a limitation in their in vitro model [1]. Furthermore, it was highlighted that the presence of the metabolites in plasma as well as in urinary excretions is of high importance during bioavailability studies involving certain food groups. Moreover, in order for the microbial metabolites to be estimated after in vivo consumption, their potential liver conjugation should be also considered due to the possible phase II enzyme action [1].

In another study, Gowd et al. [35] examined the metabolites produced after the in vitro GI digestion and human gut microbial fermentation of blackberry anthocyanins, throughout a period of 0 to 48 h, using the HPLC-UV analysis (Table 1) [35], a commonly used method to assess the solubility and absorption patterns of chemical substances [93]. The main compounds detected in the ethanolic extract of blackberry prior to GI digestion were C3G followed by a low concentration of cyanidin 3-O-dioxalyglucoside [35]. However, after GI digestion, anthocyanins were undetectable. Other (poly)phenols such as rutin, coumarin, and 2, 4, 6-trihydroxybenzaldehyde were identified in the initial blackberry ethanolic extracts but were entirely degraded after the GI digestion. Catechol was the only detectable compound, but it was not detectable after 48 h of gut microbial fermentation. Interestingly, several phenolic metabolites were formed throughout gut microbial fermentation and with simultaneous concentration change based on time, including gallic acid, *p*-coumaric acid, ferulic acid, chlorogenic acid, 3,4-dihydroxybenzoic acid, 2,4,6-trihydroxybenzoic acid, 2,4,6-trihydroxybenzaldehyde, rutin, and coumarin. The authors concluded that digestion and fermentation improved the biological activity of blackberry on the basis of the increased glucose consumption and glycogen content, as well as the palmitic acid-induced reactive oxygen species overproduction, mitochondrial membrane collapse, and glutathione depletion of the evaluated liver hepatocellular carcinoma cells [35]. This observation was similar to previous findings of Fernandes et al. [37], and the authors attributed the absence of anthocyanins to their sensitivity to pH modifications, as well as the changes these anthocyanins endured throughout the process of stimulated GI digestion. These studies showed that simple anthocyanins (C3G) are being degraded in a faster pace than the complex anthocyanins (cyanidin 3-O-dioxalyglucoside) by the gut microbiome [35].

Tomas et al. [89] aimed to assess the bioaccessibility of phenolics from blackberry purées after addition of 10% pectin and inulin through simulated in vitro GI digestion and colonic fermentation models. The authors performed targeted analysis of blackberry anthocyanins (Table 1), whereby three abundant anthocyanins, namely, C3G, malvidin 3-O-glucoside, and cyanidin were identified. UPLC coupled with Q-TOF–MS was then employed for the unbiased analysis (Table 1) of the untargeted metabolomics profile of both free and bound phenolics of blackberry purée samples [89], which enabled a complete metabolome comparison among the samples [94]. The result revealed that the predominant anthocyanin in the free phenolic fraction was cyanidin, whereas the bound phenolic fractions mainly contained glycosidic forms of cyanidin, peonidin, and petunidin [89]. A semi-quantitative approach using the UPLC–Q-TOF–MS (Table 1) further revealed that anthocyanin metabolites started to form during a period of 8, 24, and 48 h of in vitro colonic fermentation. In addition, the interaction among fibres (pectin and inulin) and blackberry purées determined a noticeable modification of anthocyanins that resulted in the formation of additional low-molecular-weight metabolites including 4-vinylphenol, benzoic acid, and tyrosol, together, as well as phenolic acids such as ferulic and gallic acid. This implied that the anthocyanins reaching the large intestine promoted the formation of low-molecular-weight compounds [89].

Blackberry in vitro-digested (poly)phenols and its enzymatically hydrolyzed major aglycones were assessed by Figueira et al. [95] to evaluate their blood–brain barrier (BBB) transport and neuroprotective potential, whereas human brain microvascular endothelial monolayers were utilized as an in vitro BBB model for a period of 2 to 24 h [95]. This type of monolayer has shown a characteristic high trans-endothelial electric resistance, and therefore they have been highly used in several studies assessing the in vitro examination of the human BBB [96]. While the in vitro digested (poly)phenol extracts mimicked the bioaccessible compounds in the GIT after PD in the bloodstream, those of the enzymatically hydrolyzed aglycones were analogous to the bioaccessible fraction produced after the occurrence of deglycosylation reactions in the intestine. After LC-multi-stage mass spectrometry (MS^n^) characterization (Table 1), the identification of the major compounds was performed after comparison with photodiode array (PDA) detector profiles, molecular weights, and their fragmentation patterns obtained from the existing literature. Glycosylated derivatives of various compounds were detected in the in vitro-digested blackberry samples, whereas in the samples of enzymatically hydrolyzed major aglycones, additional degradation products were present. For both extracts, the main metabolites that were transported across the BBB endothelium were quercetin-glucosides, myricetin, myricetin glucosides, and kaempferol. Therefore, these components exhibited a high neuroprotective potential [95].

In the study Azofeifa et al. [77], different blackberry juice samples were assessed after being subjected to in vitro digestion to indicate changes associated with the chemical structures or composition of the (poly)phenols and their subsequent effect on their antioxidant potential. The HPLC-UV analysis (Table 1) indicated that the gastric conditions slightly affected the phenolic composition, whereas major alterations were observed for the non-dialyzed and the dialyzed analysed fractions. The HPLC-UV analysis of the digested samples showed C3G and cyaniding 3-malonyl glucoside were the main anthocyanins present. On the basis of the assumption that anthocyanins were present in the non-digested sample at a concentration of 100% of the total metabolites, the non-dialyzed fraction retained 46% of them, and in the case of the dialyzed one, a low percentage of 19% was retained [77]. In vitro-digested samples of raspberry showed higher total phenolic and flavonoid content after GI digestion in the study of Chen et al. [16]. After performing LC–MS analysis (Table 1), the authors found that pelagonidin hexoside as well as additional phenolic compounds including esculin and kaempferol hexoside displayed a significantly higher concentration in the digested samples. This outcome was attributed to structural and biological activity modifications occurring as a result of pH alteration and the effect of digestive enzymes, which led to variable levels of degradation phenomena to (poly)phenols such as anthocyanins, procyanidins, flavan-3-ols, and flavones [16].

An additional study [97] assessed the in vitro total bioaccessibility of the most abundant (poly)phenols in blackberry through HPLC analysis (Table 1), utilizing a GI and colonic fermentation model. In this study, dialysis membranes were used prior to the colonic fermentation in order to achieve the simultaneous dialysis of (poly)phenols and their metabolism by the colonic microbiome, as well as to inhibit the further fermentation and the formation of carbon dioxide. C3G was again the main anthocyanin detected, constituting more than 84% of the total quantified anthocyanins. Roughly 11% of the remaining anthocyanins were composed of cyanidin 3-O-xyloside, acylated derivatives of C3G, cyanidin 3-O-(6-O-malonyl-glucoside), and cyanidin 3-O-β-(6″-(3-hydroxy-3-methylglutaroyl)-glucoside. At an intestinal level, C3G was bioaccessible at a rate of 1.8%. As much as 3.3% of the predominant (poly)phenols in blackberry were bioaccessible (including ellagitannins at the intestinal and colonic level) [97].

The research hypothesis of Olivas-Aguirre et al. [7] entailed the assumption that the first-pass metabolism of berry polyphenols could modify their final bioactivity. Therefore, the authors examined antioxidant activity and phenolic profiles of freeze-dried berries that included blackberries. Apparent permeability and first-pass biotransformation of the polyphenols using ex vivo bioanalytical system, which included everted gut rat sacs were assessed. HPLC–Q-TOF–MS was initially employed to profile the polyphenolics in 80% methanol (MeOH) extracts of blackberries (Table 1) [7]. The authors used a real-time differential pulse voltammetry (DPV) (0–120 min) to monitor the precursor polyphenols and their metabolites, as this method can provide a thorough description of the antioxidant properties of different molecules including variations in their mechanisms [98]. DPV methods with graphite and carbon electrodes have been employed in the past for the evaluation of (poly)phenols in several matrices, including wine, tea, fruit, and fruit juices, as well as olive oils [99]. According to the authors, this was the first study utilizing DPV to assess the apparent permeability and enteral biotransformation of polyphenols from berries [7]. Epicatechin (*m/z* 289.1), pelargonidin (*m/z* 272.1), cyanidin 3-O-arabinoside (*m/z* 420.2), pelargonidin 3-O-glucoside, (*m/z* 433.2), cyanidin 3, 5-O-diglucoside (*m/z* 612.4), and C3G (*m/z* 450.1) were the phenolic precursors detected in the 80% MeOH extracts of blackberries. After the first passage under simulated intestinal conditions (pH 7.0), anthocyanins were pH-unstable, with four metabolites, namely, quinic acid (*m/z* 191), chlorogenic acid (*m/z* 353), caffeic acid (*m/z* 179), and malvidin 3-O-glucoside (*m/z* 794) [7].

In order to evaluate the effect of (poly)phenols present in raspberry against oxidative stress and reduce the occurrence of microglia activation, Garcia et al. [100] used an in vitro approach to mimic the GI digestion process. In this way, the compounds that maintained their initial structure were those that would be bioaccessible after release in the serum, which were compared to those present in the non-digested raspberry extract. As indicated by HPLC–MS analysis (Table 1), a low percentage equal to 19.2% of the total (poly)phenols survived the digestion process, and this proportion was in accordance with that of previous research, as the authors indicated. Anthocyanin recoveries varied between 40 and 60%, with the higher losses being potentially related to their degradation or attachment to protein molecules, or even their segregation on the dialysis membrane of the gut model [100].

### 3.2. In Vivo Animal Models (Rat)

Alongside human clinical trials, animal studies comprise one of the most efficient methods to investigate the in vivo bioactivity of different natural compounds [88]. Hence, the majority of the existing studies are based on animal models, predominately those involving rodents [101] as a result of their high genomic and physiologic resemblance with humans [102]. Talavera et al. [36] scrutinized the anthocyanin metabolic distribution pathways in the digestive organs (stomach, jejunum, and liver), as well as in the kidney and brain of rats, which received a diet high in blackberries (15 g blackberry extract per kg diet contained 14.8 mmol anthocyanins) for a period of 15 days. The qualitative and quantitative analyses of anthocyanin metabolites were carried out through HPLC–MS/MS and HPLC with diode array detector (DAD), respectively. The findings suggested that anthocyanins such as C3G and cyanidin 3-O-pentoside were present in the stomach. As the anthocyanins passed through jejunum, liver, and kidney, methylated and glucurono-conjugated anthocyanins were detected, including peonidin and cyanidin monoglucuronides. The liver contained the highest concentration of methylated anthocyanins, whereas both jejunum and plasma contained aglycone forms. Furthermore, anthocyanins such as peonidin 3-O-glucoside could also reach the brain after an anthocyanin-rich diet, while in the urinary excretion of rats, the total anthocyanin content was negligible, possibly due to degradation to small molecules, as noted by Zhang et al. [85]. Additional studies have also suggested the potential of anthocyanin derivatives to reach the brain [103]. At the same time, due to their rapid absorption, these derivatives were detectable in the brain in a period of only 10 min after they reached the stomach [103]. In another study by Felgines et al. [84], the anthocyanin distribution in the various tissues (adipose tissue, bladder, heart, testes, and prostate), as well as in plasma and urine of rats, after a diet supplemented with a blackberry anthocyanin extract, was examined fora period of 12 days. The qualitative and quantitative analyses of the samples through HPLC–DAD (Table 1) indicated the predominance of anthocyanins in the bladder followed by prostate. The anthocyanin distribution varied among the different organs (heart, prostate, and testes), and they contained C3G and a small concentration of cyanidin monoglucuronide, whereas additional methylated derivatives were present in the adipose tissue. However, the anthocyanin precursors and their metabolites were low in the urine [84].

As has been reported in rat models after the provision of a blackberry diet, C3G is rapidly and effectively absorbed in its intact form, indicating that its small molecular weight enables its higher diffusion [77]. However, under in vivo conditions, further metabolism of the unabsorbed anthocyanins could occur through the action of the intestinal microbiota, resulting in the production of lower molecular weight compounds, including phenolic acids, which could be absorbed at the colon and promote the antioxidant capacity in the serum [77]. Gu et al. [79] determined the effect of a diet rich in blackberry 10% (w/w) freeze-dried blackberry powder, for a period of 6 weeks, on the colon microbiome of mice, after assessing the presence and the concentration of the produced metabolites in their colon and circulation. HPLC–MS/MS (Table 1) was used to initially analyse the anthocyanin metabolites and protocatechuic acid. As it was observed, a diet high in blackberries had an effect on mucosal colonic microbiota, even though a more apparent effect was shown for the luminal microflora. Protocatechuic acid was quantified using the *m/z* transition 153→109, whereas *m/z* 153→53 and 153→91 served as confirmatory qualifier transition ions. The highest concentration of the anthocyanin metabolite protocatechuic acid was found in the luminal contents (232.49 ± 31.71 μmol/kg), followed by colon tissue, plasma, liver, and prostate and concentrations of 4.46 ± 2.46, 1.24 ± 0.74, 1.12 ± 0.24, and 0.84 ± 0.10 μmol/kg, respectively [79].

### 3.3. In Vivo Human Models

As it has been suggested, it is essential to evaluate the in vivo effect and mechanisms of (poly)phenols in order to reach a satisfactory level of understanding concerning their activity and contribution to a healthy diet [29]. Although the metabolism of these compounds has been elucidated in both human subjects and various animal species, limited information is available with respect to the diversity in the physiological effects of these metabolites when present in animal models in comparison to those in humans [101]. Even if clinical trials have been conducted with human subjects after the consumption of berries such as blackberries, these studies are neither cross-sectional nor longitudinal to assess the different dietary habits alongside habitual aspects and risk factors [88]. The assessment of gut microbial composition and consistency, and its further effect on the food digestibility, has mainly centred on faecal samples that can provide significant information of the activities of the distal large intestine [92].

Felgines et al. [2] evaluated the anthocyanin derivatives of blackberries through the examination of urinary excretions of human subjects who had consumed 200 g of whole blackberries as part of a meal (960 µmol of anthocyanins expressed as C3G equivalents). Qualitative and quantitative analyses of blackberry anthocyanins and their metabolites in urinary samples were performed by HPLC coupled with ESI^+^-MS/MS, and HPLC with UV–VIS, respectively (Table 1). C3G (*m/z* 449→287), was the main anthocyanin in blackberries, whereas three additional anthocyanins were present and in relatively low concentrations, namely, cyanidin 3-xyloside (*m/z* 419→287), cyanidin malonylglucoside (*m/z* 535→287), and cyanidin dioxalylglucoside (*m/z* 593→287). Several metabolites were identified in urine, including C3G, and the rest comprised cyanidin (*m/z* 287→137), cyanidin diglucuronide (*m/z* 639→287), cyanidin monosulfate (*m/z* 367→287), cyanidin 3,5-diglucoside (*m/z* 611→287), cyanidin glucuronide (*m/z* 463→287), cyanidin sulfate (*m/z* 367→287), peonidin (*m/z* 301→201), peonidin-3-glucoside (*m/z* 463→301), peonidin 3-xyloside (*m/z* 433→301), and peonidin glucuronide (*m/z* 477→301). However, anthocyanidin monoglucuronides were the main metabolites present in urinary excretions, constituting over 60% of all metabolites [2].

**Figure 5:**
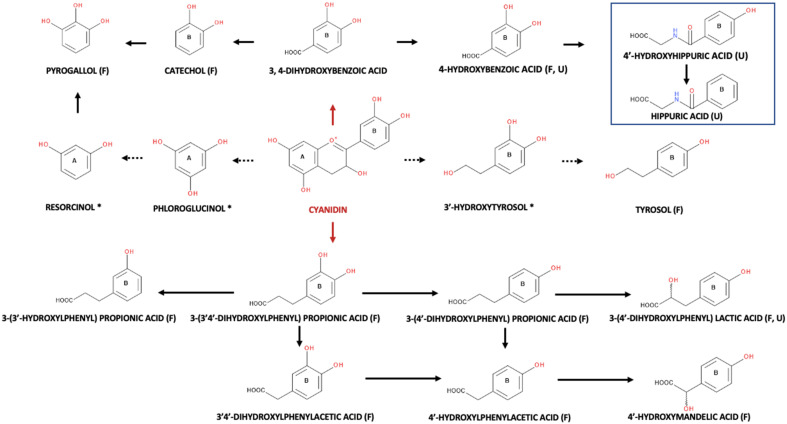
The proposed pathways for the conversion of cyanidin-related anthocyanins from red raspberry to phenolic acids from the illustration in the work of González-Barrio et al. Reprinted from ref. [40]. According to the authors, the letter F indicates the identified metabolites in faecal suspensions, U shows the catabolites present in urine, and * indicates potential intermediate products but not in detectable concentrations.

In their novel study on microbial metabolites of polyphenols following consumption of red raspberry purée (125 g/day), Zhang et al. [85] revealed that the microbial metabolites derived from anthocyanins and proanthocyanidins in urine samples were small molecular weight phenolic acids such as benzoic acids, phenylcinnamic acids, phenylvalerolactones, and phenyl acids [85]. González-Barrio et al. [40] evaluated in vivo and in vitro colonic fate of anthocyanins after consumption of 300 g of raspberries by healthy individuals, aiming to identify phenolic acid catabolites, as well as those that are excreted in the urine. GC–MS was used for the analysis of derivatized phenolic acids in urine and faecal suspensions, whereas HPLC–PDA–MS/MS was used for anthocyanins in purified samples (Table 1). However, in the cases where the target compounds were conjugates, LC was considered as a superior separation technique compared to GC, due the low volatility of these compounds. GC–MS is also deemed unsuitable for the analysis of phenolic sulphate and glucuronide metabolites [104]. As has been shown, approximately 40% of the ingested anthocyanins, and C3G in particular, passed into the large intestine. The evaluation of the in vitro anaerobic faecal suspensions showed the conversion of anthocyanins to phenolic acids, whereas that of the urinary samples indicated that after their colonic formation, a number of phenolic acids are subjected to phase II metabolism and the subsequent formation of compounds that are not present in faecal suspensions. In parallel, after assessing the effect of heat treatment on the degradation of anthocyanins in the faecal suspensions, the authors confirmed the contribution of colonic microbiome in the formation of phenolic acids. Taking into consideration the obtained data, the authors finally suggested a thorough colonic pathway for cyanidin from raspberries, which includes different conversion pathways for its B-ring to benzoic acid (protocatechuic) and phenyl acid (3-(3′, 4′)-dihydroxyphenylpropionic), with these having been anticipated as the initial metabolites (Figure 5) [40].

Targeted and non-targeted LC−MS^n^ analyses (Table 1) were used to evaluate the (poly)phenols present in ileal fluid 0–8 h post-ingestion and after the consumption of 300 g of raspberry including seeds (that were not eliminated/crushed by processing) [105]. The former approach aimed to identify the major anthocyanins at varying recoveries, whereas the later aimed to assess the Orbitrap MS produced data of shifted peaks after supplementation [105]. The use of Orbitrap MS is not surprising, as its significant contribution after coupling with LC and the subsequent provision of high mass resolving power in conjunction with tandem MS capabilities has become well known during the last few years [106]. The Orbitrap-generated MS/MS data were processed by the SIEVE software [105], an ad hoc tool that enables the quantification of different compounds as a correction pre-processing step. Hence, it enables the improvement of quantitative data through different steps including the correction of retention time, the reduction of noise, and the alignment of the chromatogram [107]. As speculated, several compounds including previously unidentified proanthocyanidin derivatives, might have been produced after raspberry seeds remained intact in ileal fluid, whereas other compounds (phenolic metabolites, anthocyanin degradation products, and unidentified compounds) were potentially produced either in the gut epithelia or after absorption into the circulatory system and their further efflux into the gut lumen. The dominant raspberry anthocyanin, i.e., cyanidin 3-O-sophoroside, was also the predominant in the ileal fluids, even though it exhibited a high variability among the human subjects (2–36%). Despite exhibiting high variability, other low concentrated anthocyanins exhibited higher mean recoveries in several cases in the following order: cyanidin 3-O-(2″-glucosyl)-rutinoside (28.6%) > pelargonidin 3-O-sophoroside (24.0%) > cyanidin 3-O-sophoroside (21.7%) > cyanidin 3-O-rutinoside (9.2%) > C3G (1.3%) [105]. However, as it has been recently shown, the quantification of polyphenols in biological samples can be underestimated. This is attributed to the fact that various catabolism-derived (poly)phenol metabolites can be also absorbed into the blood circulation and are not estimated. Subsequently, a high number of these components can be subject to further metabolism and be conjugated by phase II enzymes, resulting in glucuronidated, sulphated, and methylated compounds [61].

**Table 1:** Identification and quantification analytical techniques of anthocyanins and their metabolites in blackberries and raspberries.

Compounds/Samples	Chromatography	Detection System	Chromatographic Conditions	Reference(s)
	**Identification/Quantification**			
Anthocyanins in Blackberries/Extracts of 0.12 mol/L hydrochloric acid (HCl) in MeOH	HPLC	DAD-UV/Vis	Column: Hypersil C18 (4.6 × 150 mm^2^, 5.0 µm) Guard Column: Hypersil C18 (10 × 4 mm2, 5 µm) Eluents: (A) 1.0% Phosphoric acid (H_3_PO_4_) in H_2_O; (B) Acetonitrile (ACN) Gradient: 100–90% A for 10 min; 90–75% A for 30 min Run (t): 40 min Flow rate: 1.0 mL/min Detection wavelength: 524 nm	[2]
	**Identification/Quantification**			
Anthocyanins in Human urine after Blackberries ingestion/Acidified samples with 0.2 mol/L HCl	HPLC	ESI^+^-MS/MS	Column: Hypersil BDS C18 (2.1 × 150 mm^2^, 5.0 µm) Eluents: (A) (5:2:93) ACN: Formic acid (HCOOH): H_2_O; (B) (40:2:58) ACN: HCOOH: Water (H_2_O) Run (t): 40 min Flow rate: 0.2 mL/min Internal Standard: Cyanidin 3,5-diglucoside	
	**Identification**			
Sugar-free Blackberry/Extracts of 80% MeOH	HPLC	ESI^−/+^-Q-TOF-MS	Column: Zorbax Eclipse Plus C18 (2.1 × 50 mm2, 1.8 µm) Guard Column: Same packing Eluents: (A) 0.1% HCOOH in H_2_O; (B) ACN Run (t): 12.5 min Flow rate: 0.4 mL/min MS scan: 100–1000 *m/z*	[7]
	**Untargeted Identification/Semi-Quantification**			
First-pass metabolism of phenolic compounds of Blackberries/Ex vivo first-pass metabolism biosystem	HPLC	ESI^−/+^-Q-TOF-MS	As for identification, under the recommendations of Koistinen et al. [45]	
		The subsequent considerations were based on the real-time oxidation/reduction phenomena in the employed ex vivo system: *m/z* ± 0.3 (mass accuracy), [M-H]^+^ (Anthocyanins; rutin) [M-H]^−^ (All other polyphenols)	Semi-Quantification: Ion abundance at t_0_ and t_120_ of parent polyphenols and their metabolites	
	**Identification/Quantification**			
Phenolic compounds in Blackberries/After in vitro digestion of Blackberries; After colonic fermentation of Blackberries	HPLC	DAD	Column: Promosil C18 (4.6 × 250 mm^2^, 5.0 μm) Eluents: (A) 1.5% HCOOH in H2O; (B) 0.1% HCOOH in ACN Gradient: 5–13% B (0–15 min); 13–15% B (15–20 min); 15–30% B (20–25 min); 30% B (25–28 min); 30–45% B (28–32 min); 45% B (32–35 min); 45–90% B (35–40 min); 90% B (40–45 min); 90–5% B (45–55 min); 5% B (5 min) Run (t): 60 min Flow rate: 0.8 mL/min Injection volume: 10 μL Detection wavelength: 280 and 520 nm	[35]
	**Identification and Quantification**			
Derivatized phenolic acids in urine and faecal suspensions	GC	Dual-Stage Quadrupole (DSQ)^+^-MS	Injection volume: 1 μL in split mode (25:1). Inlet T: 220 °C Oven T: 40–160 °C at 20 °C/min; 160–200 °C at 1.5 °C/min; 200–250 °C at 10 °C/min; 250–300 °C at 40 °C/min, held for 5 min. Transferred line T: 310 °C Gas: Helium Flow rate: 1.2 mL/min	[40]
	**Identification and Quantification**			
Anthocyanins, ellagic acid, punicalagin, and urolithins in urine and purified faecal suspensions	HPLC	PDA-ESI-MS/MS [M-H]^+^ (Anthocyanins); [M-H]^−^ (Ellagitannins, ellagic acid and urolithins)	Column: Phenomenex Synergi RP-POLAR 80Å (4.6 × 250 mm^2^, 4.0 μm) Eluents: (A) 0.1% HCOOH in H2O; (B) MeOH Gradient: 10–40% B (60 min) for anthocyanins; 10–65% B (50 min) for punicalagin, ellagic acid and urolithins Run (t): 60 min for anthocyanins; 50 min for punicalagin, ellagic acid and urolithins Flow rate: 1.0 mL/min T: 40 °C MS scan: 150–1500 *m/z* Wavelength for urolithins: 305 nm	
	**Identification**			
Phenolic Compounds of Blackberries/Supernatant of fresh fruits acidified with 1% HCOOH (*v/v*)	HPLC	ESI^−/+^-MS/MS [M-H]^+^ (Anthocyanins); [M-H]^−^ (All other polyphenols	Column: Atlantis dC18 (2.1 × 150 mm^2^, 3 μm) Eluents: (A) 1% HCOOH in H2O; (B) Acetonitrile (ACN) Gradient: 5–40% B for 40 min for all the phenolic compounds except for anthocyanins (5–35% B for 30 min) Run time (t): 40 min for all the phenolic compounds except for anthocyanins (30 min) Flow rate: 0.17 mL/min	[1]
	**Identification**			
Metabolites after Faecal Fermentation of Blackberries/Samples after 5 and 24 h	HPLC	ESI^−/+^-MS/MS [M-H]^+^ (Anthocyanins); [M-H]^−^ (All other polyphenols)	Column: Atlantis dC18 (2.1 × 150 mm^2^, 3 μm) Eluents: (A) 1% HCOOH in H_2_O; (B) Acetonitrile (ACN) Gradient: 0–40% B (Initial rough identification); 0–35% B (Multiple reaction monitoring (MRM) method) Run (t): 35 min (Initial rough identification); 10 min (MRM method) Flow rate: 0.17 mL/min	
	**Identification/Quantification**			
Protocatechuic acid in extracts of mouse plasma, liver, prostate, colon tissue, and luminal content after consumption of black raspberries	UPLC	TQD-MS/MS	Column: Zorbax Eclipse Plus HD C18 (2.1 × 50 mm^2^, 1.8 μm) Eluents: (A) 0.01% acetic acid in H_2_O; (B) 0.01% acetic acid in MeOH Gradient: 0% B (0.5 min); linear increase to 95% B (5 min); 0% B (6.5 min) Run (t): 6.5 min Flow rate: 0.4 mL/min; T: 40 °C	[79]
	**Identification**			
*In vitro* digested blackberry juice samples	HPLC	DAD-ESI^−/+^- Ion Trap (TRAP)- MS/MS	Column: Lichrospher ODS-2 (4.6 × 250 mm^2^, 5.0 μm) Eluents: (A) 0.1% HCOOH in H2O; (B) ACN: H2O: HCOOH (80:18:2 *v/v/v*) Gradient: 5-25% B (50 min); 25–100% B (10 min); wash (5 min); equilibration (15 min); 0% B (0.5 min); linear increase to 95% B (5 min); 0% B (6.5 min) Run (t): 92 min Flow rate: 0.5 mL/min Detection Wavelength: 200–600 nm MS scan: 100–2000 *m/z*	[77]
	**Quantification**			
	HPLC	DAD-ESI^−/+^-TRAP- MS/MS	Column: Lichrospher ODS-2 (4.6 × 250 mm^2^, 5.0 μm) Eluents: (A) 0.1% orthophosphoric acid in H2O; (B) 0.1% orthophosphoric acid in MeOH Gradient: 0–30% B (3 min); 30–50% B (8 min); 50–70% B (12 min); 70–80% B (17 min); wash with 100% of B (2 min); equilibration for 15 min prior to each analysis Run (t): 34 min Flow rate: 1.0 mL/min Quantification Wavelength: 254 nm	
	**Identification/Quantification**			
Anthocyanins after Blackberries ingestion in Rat urine, plasma, bladder, prostate, heart, testes and adipose tissue	HPLC	DAD-UV/Vis	Column: Uptisphere 3 ODB C18 (4.6 × 150 mm^2^, 3.0 μm) Guard Column: Uptisphere 3 ODB C18 (10.0 × 4.0 mm^2^, 5 μm) Eluents: (A) 1.0 % H3PO4 in H2O; (B) ACN Gradient: 100–90% A for 10 min; 90–75% A for 30 min Run (t): 40 min Flow rate: 1.0 mL/min Detection wavelength: 524 nm Internal Standard: Cyanidin 3,5-diglucoside	[84]
	**Identification**			
Polyphenols in Red Raspberry and their metabolites in human biological samples (plasma, urine, breast milk)	UPLC for raspberry polyphenols/metabolites; not including phenolic acids	Q-TOF-ESI^−/+^-MS/MS [M-H]^+^ (Anthocyanins); [M-H]^−^ (Ellagitannins, flavon-3-ols, urolithins and other analytes)	Column: RP Poroshell C18 StableBond (2.1 × 150 mm^2^, 2.7 μm) Eluents: (A) 0.1% HCOOH in H2O; (B) 0.1% HCOOH in ACN Gradient: 5% B; 5–15% B (10 min); 15–20% B (12 min); 20–50% B (20 min); 50–90% B (23 min); 5% B (25–30 min) Temperature (T): 35 °C Run (t): 30 min Flow rate: 0.3 mL/min Injection volume: 1.0 μL for raspberry and 5.0 μL for biological samples	[85]
	**Identification**			
	UPLC for phenolic acids and derivatives in biological samples	Q-TOF-ESI--MS/MS	Column: Pursuit 3 PFP (2.0 × 150 mm^2^, 3.0 μm) Eluents: (A) 0.1% HCOOH in H2O; (B) 0.1% HCOOH in ACN Gradient: 5% B; 5–10% B (3 min); 10–15% B (7 min); 15 B (9 min); 15–20% B (10 min); 20% B (10–11 min); 20–25% B (12 min); 25–30% (13 min); 30% (13–14 min); 30–95% (15 min); 5% (16–20 min) T: 40 °C Run (t): 20 min Flow rate: 0.4 mL/min Injection volume: 5.0 μL	
	**Quantification**			
	UPLC	TQD Same ESI conditions with Q-TOF	Same Separation Conditions with Q-TOF and dynamic multiple re-action monitoring mode	
	**Targeted Identification**			
Anthocyanins in *in vitro* digested blackberry purée	HPLC	PDA	Column: Supelcosil C18 (4.6 × 250 mm^2^, 5 μm) Eluents: (A) 0.1% trifluoroacetic acid (TFA) in H2O; (B) 0.1% TFA in ACN Gradient: 5% B (0 min); 35% B (0–45 min); 75% B (45–47 min); 5% B (47–54 min) Run (t): 54 min	[89]
	**Untargeted Identification/Semi-Quantification**			
Free and bound polyphenols in undigested matrices (black-berry purée)In vitro fermented samples af-ter the addition of in vitro digested blackberry purée	UPLC	ESI^+^-Q-TOF-MS	Column: Agilent Zorbax Eclipse-plus (2.1 × 75 mm^2^, 1.8 μm) Eluents: (A) 1.5% HCOOH in H2O; (B) 0.1% HCOOH in ACN Gradient: 6–94% B Run (t): 33 min Flow rate: 0.2 mL/min MS scan: 50–1200 *m/z*	
	**Identification**			
Blackberry *in vitro* digested (poly) phenols and its enzymatically hydrolysed major aglycones	HPLC	Linear ion trap (LTQ) Orbitrap-MS^n^	Column: Phenomenex Synergi RP18 (2.0 × 150 mm^2^, 4.0 μm) Guard Column: Phenomenex Security GuardTM guard (2.0 × 4.0 mm^2^) Eluents: (A) 0.1% HCOOH in H2O; (B) 0.1% HCOOH in ACN Gradient: 98% A to reach 5% B (5 min); 35% B (25 min); 100% B (26–29 min); 2% B (30 min) Run (t): 30 minFlow rate: 0.2 mL/min	[95]
	**Identification/Quantification**			
*In vitro* digested wild raspberry extracts	UPLC	UV-ESI^−^-TOF-MS/MS	Column: Promosil C18 (4.6 × 250 mm^2^, 5.0 μm) Eluents: (A) MeOH; (B) 0.1% HCOOH in H2O Gradient: 5–14.2% A (20 min); 14.2–60% A (50 min); 60–5% (10 min); isocratic elution (5 min) Run (t): 92 min Flow rate: 0.8 mL/min Detection Wavelength: 260 nm MS scan: 100–2000 *m/z*	[16]
	**Identification/Quantification**			
Anthocyanins from blackberry at simulated GI and colonic levels	HPLC	UV-PDA	Column: Phenomenex Gemini (4.6 × 250 mm^2^, 5.0 μm) Eluents: (A) 5.0% HCOOH in H2O; (B) MeOH Gradient: 10% B (0 min); 15% B (5 min);20% B (15 min); 25% B (20 min); 30% B (25 min); 60% B (40 min), 10% B (42 min); 10% B (45 min) Run (t): 45 min Flow rate: 1.0 mL/min Detection Wavelength: 520 nm T: 30 °C Quantification of anthocyanins: C3G	[97]
	**Identification/Quantification**			
Digested and non-digested raspberry extracts	HPLC	ESI^+/−^-MS	Column: Synergi Hydro C18 (2.0 × 150 mm2,.0 μm) Eluents: (A) 0.1% HCOOH in H_2_O; (B) 0.1% HCOOH in ACN Gradient: 5% B (0 min) to 40% B (30 min) Run (t): 30 min Flow rate: 0.2 mL/min	[100]
	**Targeted Analysis**			
Raspberry phenolics in ileal fluids	HPLC	PDA-ESI^+/-^-MS/MS [M-H]^+^ (Anthocyanins); [M-H]^−^ (Ellagitannins, ellagic acid and urolithins)	Column: Synergi RP-POLAR 80 Å (4.6 × 250 mm^2^, 4.0 μm) Eluents: (A) 0.1% HCOOH in H2O; (B) 0.1% HCOOH in MeOH Gradient: 10–40% B (60 min) Run (t): 60 min Flow rate: 1.0 mL/min, split to 0.3 mL/min after passing DAD	[105]
			Quantification Anthocyanins (520 nm) as C3G equivalents; Ellagitannins (260 nm) as punicalagin equivalents; Ellagic acid and its conjugates (365 nm) as ellagic acid equivalents	
	**Untargeted Analysis**			
	HPLC	PDA-ESI^−^-MS/MS	Column: Synergi RP-POLAR 80 Å (4.6 × 250 mm^2^, 4.0 μm) Guard Column: Phenomenex (4.0 × 2.0 mm^2^) Eluents: (A) 0.1% HCOOH in H2O; (B) 0.1% HCOOH in ACN: H2O (1:1 *v/v*) Gradient: 5% B (0–4 min); 5–50% B (4–22 min); 50–100% B (22–32 min) Run (t): 60 min Flow rate: 0.3 mL/min Detection Wavelength: 280, 325, 365, and 520 nm T: 30 °C MS scan: 80–2000 *m/z*	

## 4. Ellagitannins and Their Metabolites in Blackberries and Raspberries

Ellagitannins are (poly)phenolic compounds present in significant concentrations in blackberries and raspberries [11,108]. These complex compounds, which are characterized by high molecular weights (up to 4000 dalton (Da)), are composed of hexahydroxydiphenic acids forming diesters with sugars predominately with β-D-glucose [6]. Both raspberries and blackberries share similar major ellagitannins, namely, lambertianin C and sanguiin H-6 [109,110,111]. However, their high molecular weights of 2804 and 1869 Da, respectively, as well as their low solubility have been known to hamper their bioavailability [111,112]. Accurate structural elucidation and quantification of ellagitannins and ellagic acid conjugates in different matrices are important for assessing their effect on human health. Unfortunately, their identification and analysis are impeded by the absence of pure standards commercially, as well as in methods that have the capacity to sustain the complex polymer in its native form [12]. Therefore, the usual exploitation of precursor compound as analytical standard for quantitative purposes of its metabolite, which is structurally different and entails a high possibility of producing incorrect results [49]. Nonetheless, in the case of urolithins, studies during recent years tended to utilize either chemically synthesized compounds or those isolated from gut microbiota as well as human urine [113,114,115]. At the same time, additional barriers in the assessment of ellagitannins as well as of other (poly)phenols and their metabolites are the various methodologies used in different research studies that in parallel to the great number of metabolites result in difficulties associated with both their collection and organization [44].

**Figure 6:**
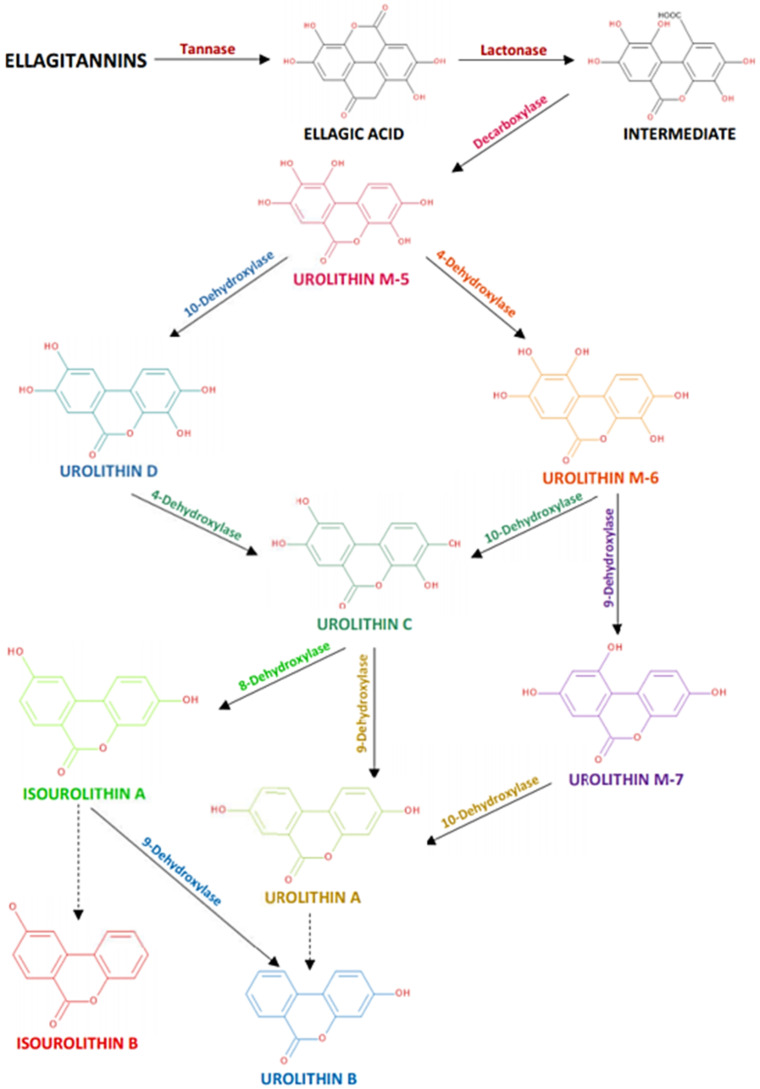
The effect of different dehydroxylases on the microbial biotransformation of ellagitannins to urolithins as adapted with light modification by the study of Sallam et al. [123], which originated from the study of Epsin et al. [118]. Reprinted from ref. [123].

Ellagitannins yield ellagic acid (monomeric unit) upon hydrolysis with acids or bases [6]. However, in vitro digestion studies have indicated that ellagitannins are in most cases considerably stable under the acidic conditions (pH 1.8–2.0) of the stomach, where the enzymes are incapable of decomposing or hydrolyzing them to ellagic acid units [116]. As they are hydrolyzable tannins, their absorption starts in the small intestine, mainly in the jejunum, after hydrolyzation to ellagic acid [117]. Ellagitannins and their metabolite ellagic acid have exhibited apparent biological activities in studies involving either human or animal model systems, indicating their potency against chronic diseases (i.e., cancer and cardiovascular diseases) [118]. Even though their absorption starts in the small intestine, high concentrations of ellagitannins and ellagic acid derivatives can survive digestion and enter the colon [105]. Interestingly, the further microbial metabolism of ellagic acid after the dietary intake of ellagitannins can result in the formation of a different class of metabolites known as urolithins (A, B, C, and D) [118,119,120]. Urolithins are compounds of the 6H-dibenzo-[b, d] pyran-6-one family and have various phenolic hydroxylation patterns. From a chemical perspective, urolithins constitute a combination of coumarin and isocoumarin [118]. As urolithin metabolites exhibit distinctive UV spectra, they can be detected and identified by HPLC coupled with UV/DAD, with lambda max ranging from 246 to 367 nm [119]. Animal studies have shown that the formation of these metabolites is initiated in the small intestine, suggesting that the anaerobic bacteria is responsible for this biotransformation [121]. It has been suggested that the production of urolithins stems from the removal of either one or two lactone groups after the lactonase and decarboxylase activity and the subsequent loss of hydroxyl group catalysed by a dehydroxylase [118,122]. The detailed microbial biotransformation of ellagitannins to urolithins and the effect of different dehydroxylases has been previously included in the study of Sallam et al. [123] on the basis of the previous study of Epsin et al. [118] (Figure 6).

The production of systematically absorbed urolithins has been considered as the link among the in vivo biological action of ingested ellagitannins and the questionable extent of their bioavailability [118]. After their production, urolithins are initially present in faeces as glucuronides and can be absorbed and circulated in plasma prior to their accumulation in urine as glucuronide and sulphate conjugates [124,125]. Urolithin A and B aglycones are conjugated in the liver or intestinal cells, through the action of uridine 5’-diphospho (UDP)-glucuronosyltransferases, which enhance their solubility and therefore excretion in urine [40,121]. The high bioactivity of these compounds has been also confirmed as they were present in urine even after seven days post-ingestion. The urolithins can also be directly excreted in faeces in their aglycone forms [124]. The phenotypic variability of urolithins after the metabolism of ellagitannins and ellagic acid has been related not only to the diverse food matrices [76], but also to the host colonic microbiome [76,112], with the latter being highly differentiated among either different individuals or in the same person throughout different development stages and health conditions [33]. In fact, these differences have been associated with differences in production time, amount, and forms of urolithins absorbed and excreted in urine [76]. An inherent limitation in the comparison of different studies and the evaluation of the different metabolic pathways of different (poly)phenolic compounds, such as urolithins, is the focus of different research groups on certain phenolic groups and types. Therefore, the exclusion of polyphenols present in lower concentration in food matrices from the scope of these studies can result in the detection of different metabolites in samples, even if the same food source had been used [33].

It is known that intestinal microbiota play a crucial role in the metabolism of ingested compounds [126]. Berry compounds can regulate gut microbiome through the increase or decrease of certain genera. As it has been shown, these alterations in a healthy gut can exert positive effects, whereas in the opposite case, they can lead to a better gut composition [78]. However, even if the formation of some intermediate urolithins has been associated with the presence of definite intestinal microbial species, namely, *Gordonibacter urolithinfaciens* and *Gordonibacter pamelaeae*, which belong to *Eggerthellaceae* family, the exact microorganisms that are related to the total biotransformation of ellagic acid into the final urolithins are still unidentified [127]. At the same time, even if the formulated microbial (poly)phenol metabolites have been reported in both in vitro and in vivo studies, it is important to highlight that further research is still required to associate them with clinical health effects [44].

### 4.1. In Vitro Models

Notable differences in the formation of metabolites have been observed after assessing the in vitro colonic bioconversion of polyphenols from different plant sources (grape juice, red wine, and black tea), whereas these differences were ascribed to the effect of the diverse gut microbiota [128]. The complexity of urolithin formation, and their absence after in vitro fermentation of blackberries, has been observed in the study of Dall’Asta et al. [1]. The authors attributed these findings to either the high variability in urolithin formation or to the fact that polyphenols have to initially be transformed in the small intestine in certain forms that can be further modified by the colonic microbiome [1]. A recently described study of Tomas et al. [89] indicated the presence of urolithin C as an important discriminant marker during in vitro fermentation, and as function of 5% inulin addition. On the other hand, the absence of additional urolithin metabolites was potentially ascribed to the bacterial composition of the utilized faecal pig inoculum in their study [89]. The stability and transformations of the main raspberry ellagitannins, namely, lambertianin C and sanguiin H-6, were assessed by Sójka et al. [109]. The concentration of the evaluated ellagitannins and their degradation products in aqueous media were evaluated through HPLC–DAD and LC–MS (Orbitrap), respectively. As was indicated, both lambertianin C and sanguiin H-6 exhibited good stability in acidic environments, even if they degraded rapidly degraded in neutral and slightly basic conditions and at temperatures among 60 and 80 °C. In parallel, in slightly acidic environments and pH equal to 6, the assessed ellagitannins were hydrolyzed to different intermediate products, namely, sanguiin H-2 and H-10 isomers, as well as galloyl-hexahydroxydiphenoyl-glucose isomers, whereas the main final products were composed of ellagic and gallic acids. It was further concluded that oxidation processes led to the development of compounds containing a hehydrohexahydroxydiphenic acid group, in parallel to its hydrolysis product, namely, brevifolin carboxylic acid [109].

The results of Azofeifa et al. [66], as previously analysed in Section 3.1 and Table 1, suggested that even if blackberry polyphenols can maintain their antioxidant capacity after passage through the GIT, the dialyzed fraction of blackberry juices undergo a reduction of its antioxidant potential, potentially due to the absence of ellagitannins. The HPLC analysis indicated that lambertianin C and sanguiin H-6 were the main ellagitannins in the digested blackberry juice samples. On the basis that the content of ellagitannins in the non-digested sample was 100% of the total metabolites, the non-dialyzed fraction of this study maintained 66% of their total, whereas the dialyzed fraction maintained only 6.8%. On the basis of the assumption that the utilized dialysis model could reproduce the in vivo conditions to some extent, the authors suggested that the blackberry ellagitannins are predominately retained in the colon, whereas anthocyanins exhibit a better absorbability by the cells [66]. Van de Velde et al. [81] also assessed the in vitro bioaccessibility of ellagitannins from blackberry through HPLC (Table 2), except for that of anthocyanins (Section 3.1). As indicated, the intestinal bioaccessibility of ellagitannins was lower than 1% for the main ellagitannins present, namely, lambertianin C and sanguiin H-6, wherein the latter was the most abundant one. Finally, with respect to ellagic acid, its intestinal bioaccessibility was found to be 14.9% as a result of its release after ellagitannin degradation. Nonetheless, a lower percentage of roughly 10% was bioaccessible at a colonic level as a result of the hindrance of its further metabolization by the dialysis process [81].

The previously referred study of Garcia et al. [89] (Section 3.1 and Table 1) additionally showed both lambertianin C and pedunculagin-related compounds (*m/z* 783) were not present in the GI-bioaccessible fraction of raspberry. Sanguiin H-6 was present at a level of 20% compared to the non-digested raspberry, whereas sanguiin H-10 exhibited a 100% recovery. As shown previously by Van de Velde et al. [81] for blackberry, a low in vitro recovery of ellagitannins was observed, whereas Garcia et al. [89] suggested that these compounds can be highly susceptible to degradation under either acidic or basic environments. As they further suggested, there is a tendency in the degradation of higher molecular weight compounds under these conditions, such as lambertianin C, which can form sanguiin H-6 and subsequently sanguiin H-10 through the consecutive loss of ellagic acid. Hence, this could potentially support the high recovery of sanguiin H-10. Other compounds, including ellagic acid and ellagic acid 4-acetyl pentoside, exhibited recoveries of 200% and 90%, respectively. Moreover, in conjunction to the anthocyanin bioaccessible fraction that was previously analysed, raspberry bioaccessible (poly)phenols exerted a profound effect against neuronal oxidative stress, whereas they were also capable of inhibiting microglial pro-inflammatory activation [89].

### 4.2. In Vivo Animal Models

González-Barrio et al. [119] identified the bioavailable ellagitannin metabolites in humans and different animals (insects, birds, squirrels, rats, mice, pigs, beavers, sheep, bull calves) after intake of blackberries for two weeks through HPLC–UV, and HPLC–MS/MS analyses, and in particular high-resolution HPLC–Q-TOF–MS and ion trap MS/MS. These authors showed that the animals produced urolithins and that their glucuronyl and sulfate derivatives were the principal metabolites present in urine and plasma samples. In this same study, unconjugated urolithins were found in faecal, ruminal, and beaver castoreum samples. An interesting finding of the study was the diverse urolithin hydroxylation configurations that were detected in different species, suggesting that the microbiota fauna of each animal species have different dehydroxylases for removing specific hydroxyl groups from ellagic acid [119].

The study of Gu et al. [79], as described in Section 3.2 and Table 1, showed that urolithin A was the predominant metabolite of ellagitannins produced in mice after a blackberry rich diet. This metabolite was additionally the highest in concentration in luminal contents (29.14 ± 9.42 μmol/kg) in comparison to plasma (0.09 ± 0.03 μmol/kg), liver (0.35 ± 0.09 μmol/kg), prostate (0.15 ± 0.09 μmol/kg), and finally colon tissue (3.93 ± 1.21 μmol/kg). Urolithins were analysed in positive ESI mode with the following MS/MS transitions: *m/z* 229→128), 157 (urolithin A), *m/z* 213→115, 141 (urolithin B), *m/z* 245→155, 183 (urolithin C), 261→171, 199 (urolithin D), 243→171, 184 (methyl urolithin A), 259→183 (methyl urolithin C), and 257→198 (dimethyl urolithin A). Urolithin C was found in all the assessed tissues, except for prostate, whereas its concentration in colon was 45 times higher than in plasma. At the same time, urolithins B and D were not present in plasma or tissues, but they displayed 400–1000 times lower concentrations in luminal tissues in comparison to urolithin A. Finally, while comparing the produced anthocyanin and ellagitannin metabolites, the authors found that protocatechuic acid displayed a higher content than urolithins [79].

### 4.3. In Vivo Human Models

García-Muñoz et al. [39] examined the presence of urolithin A and B derivatives in urine samples of human volunteers after ingestion of 250 mL of tropical highland blackberry juice. The use of UPLC–DAD coupled with Q-TOF–MS/MS analysis (Table 2) enabled the tentative identification of 15 additional ellagitannin metabolites, namely, urolithins A, B, C, D; and M5, isourolithins A, B, C; and glucuronides, methyl urolithin A, dimethyl-urolithin C, hexahydroxydiphenyl, as well as ellagic acid, its dimethyl ether glucuronide, and dimethyl ether. The main ellagitannin derivatives present in human urine were urolithin A and B glucuronides. The same study showed three distinctive groups comprising those that excreted no or low concentrations of urolithins, those with mainly urolithin A excretions, and those who principally excreted urolithin B. However, a high variability in the quantified urolithin excretions amongst different individuals was observed despite the fact that the excretion pattern was constant for the same individual. This variability was attributed to several factors including synergistic effects among the gut microbiota, compositional variations, and inherent differences in the host endogenous excretory system. These authors also reasoned the comparatively lower hydrophobicity of urolithins C, D, M5, hexahydroxydiphenyl, and ellagic acid derivatives compared to urolithins A and B was the main reason for their low bioavailability in the intestinal lumen [39]. Differences in the production of urolithins by humans had been reported in an earlier study, wherein the authors indicated the action of different gut microorganisms influence the urolithin production [121]. After assessing human breast milk samples, Zhang et al. [85] revealed that urolithins were the most significant metabolites identified among them. In addition, the assessment of urine samples revealed the presence of three urolithins, namely, urolithin A, urolithin B, and urolithin A glucuronide [85].

A large inter individual variability in (poly)phenol metabolism was also found in the in vivo assessment of intake biomarkers after the consumption of black raspberry products intended for clinical trials [129]. These results, which corroborated with those on the previously mentioned study of García-Muñoz et al. [39], highlighted that the factors such as the genetic background of the host, as well as its gut microbiome, are significant in elucidating the bioavailability of certain components and their metabolic pathways [129]. This clinical study assessed the effect of two different types of blackberry-rich products (confection or nectar, with doses of 10 or 20 g, respectively, and for a period of 4 weeks) on metabolite production in men with prostate cancer. The performance of UPLC–MS/MS (Table 2) indicated that the excretion of metabolites was analogous to the dose received by the subject, even though it was not homogenous among them. Prior to analysis, the chemical identity of all the evaluated compounds was confirmed after the assessment of hyphenated high-resolution mass spectrometry (HRMS) and NMR data [129]. While HRMS is tending towards being one of the most frequently utilized ultra-sensitive analytical methods, the combination of NMR and MS enables the acquisition of complementary information and the expansion of metabolomics coverage [130], whereas NMR also constitutes a high reproducible method [107]. Nonetheless, in comparison to MS individually, NMR has a lower sensitivity, and therefore a lower capacity for the qualitative and quantitative analysis of metabolites. Hence, the NMR can result in failure to identify potentially significant components that are present at lower concentrations, which can be masked by larger chromatographic peaks [107]. Confection consumption led to the excretion of higher concentrations of urinary metabolites, namely, urolithin A and dimethylethylamine, indicating that food matrix is crucial with respect to colonic microbiome exposure [129].

Besides anthocyanin metabolites (Section 3.3 and Table 1), McDougall et al. [105] also detected the predominant ellagitannins present in ileal samples after supplementation of raspberries. Similarly to anthocyanins, a large inter-individual variability (1−64%) was observed for the most abundant compound of this family, namely, sanguiin H-6. In parallel, sanguiin H-10 was in most cases recovered at higher concentrations (1−31%) compared to lambertianin C (0−35%). Nonetheless, a high variability among individuals was observed, which has been highlighted also by previously analysed study [28]. As the authors suggested, the high concentrations of these compounds in ileal fluid may indicate their high stability in the GIT. However, some compounds may arise from the degradation of higher molecular weight ellagitannins, as for instance sanguiin H-6 from lambertianin C. Finally, even if ellagic acid pentoside concentration exhibited in general a low recovery (11.0−50.0%), ellagic acid was present in the ileal fluids of all individuals at elevated concentrations compared to its initial concentration in raspberries (17−304%). Hence, these higher levels may have potentially arisen from ellagitannin and/or ellagic acid pentoside degradation and the subsequent release of ellagic acid [105].

**Figure 7:**
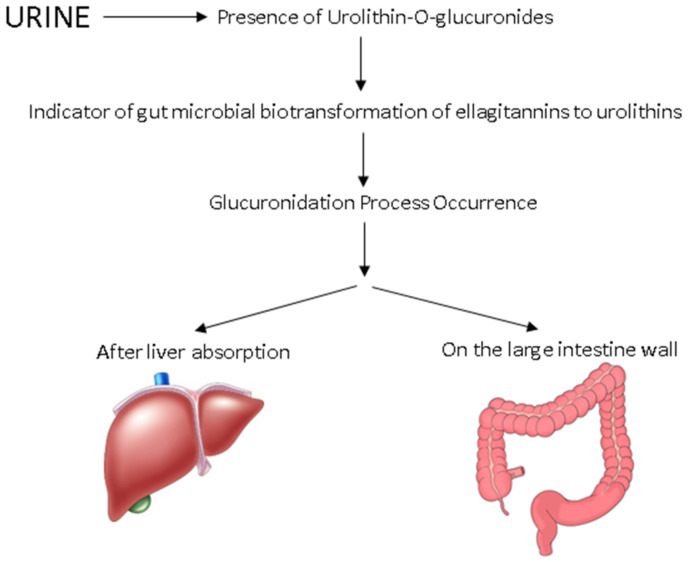
Hypothesis of in vivo gut microbial biotransformation of ellagitannins to urolithins proposed by González-Barrio et al. [40].

The previously reported study of González-Barrio et al. [40] (Section 3.3 and Table 1) evaluated besides anthocyanins, the colonic fate of ellagitannins. In this case, the in vitro anaerobic incubation of ellagitannins with faecal suspensions showed the conversion of ellagic acid into various urolithins at a level of 80% of its total content. Punicalagin was converted to ellagic acid, urolithin A, urolithin C, and isourolithin A, while a high variability in the profile was observed among the different subjects. This observation showed the inherent variability that can be observed among different individuals as a result of their colonic microbiome diversity. In addition, as urolithins were detected in vivo in urine as O-glucuronides and not aglycones, it was indicated that the colon microbiome converted ellagitannins to urolithins, whereas glucuronidation took place either on the large intestine wall and/or after absorption in the liver (Figure 7) [40]. However, it has been suggested that the metabolic capacity of the liver being approximately 100 times lower than that of the intestinal microbiota is a result of the diverse and complex nature of the latter [126].

Istas et al. indicated through a double-blind randomized control trial (10 male subjects) that the plasma urolithin metabolites after red raspberry consumption could be related with beneficial effects with respect to the endothelial function [11]. A flow-mediated dilation after 2 and 24 h [11] was used as a non-invasive technique to assess the endothelial function of the human subjects [131] after the consumption of 200 g and 400 g of red raspberries with a total (poly)phenol content of 201 or 403 mg, respectively, or a matched control drink. The analysis of raspberry (poly)phenol metabolites was performed in plasma and urinary samples by utilizing UPLC–Q-TOF–MS (Table 2), where 15 different ellagitannin metabolites comprising ellagic acid and its derivatives; isourolithin A; and urolithins A, B, and C, as well as their glucuronide and sulphate conjugates were detected [11]. This was shown for the first time that the consumption of red raspberries has the potential to augment the endothelial function in healthy human subjects for a period of 24 h, wherein the endothelial function was attributable to the form and concentration of the ellagitannin metabolites in circulation.

**Table 2:** Identification and quantification analytical techniques of ellagitannins and their metabolites in blackberries and raspberries.

Compounds/Samples	Chromatography	Detection System	Chromatographic Conditions	Reference(s)
	**Identification/Quantification**			
Plasma and urine polyphenol metabolites after raspberry drink consumption by human subjects	UPLC	Q-TOF-ESI-MS	Column: Zorbax Eclipse Plus RRHD (50 × 2.1 mm^2^, 1.8 μm) Guard column: Eclipse Plus (5 × 2.1 mm^2^, 1.8 μm) Eluents: (A) 0.1% HCOOH in H2O; (B) 0.1% HCOOH in ACN Gradient: 1% B (0 min); 10% B (5 min); 25% B (8 min); 99 % B (9.1 min); 99% B (10 min); equilibration with 1% B (12 min) Run (t): 10 min Flow rate: 0.4 mL/min	[11]
	**Identification/Quantification**			
Urolithins in plasma after raspberry drink consumption by human subjects	UPLC	Q-TOF-MS	Column: Poroshell 120 EC-C18 (100 × 3.0 mm^2^, 2.7 μm) Eluents: (A) 0.1% HCOOH in H_2_O; (B) 0.1% HCOOH in ACN Gradient: 5% B (0 min); 18% B (7 min); 28% B (17 min); 50% B (22 min); 90% B (27–28 min); re-equilibration (29 min); isocratic conditions up to 33 min Run (t): 33 min T: 25 °C Flow rate: 0.5 mL/min	
	**Identification/Quantification**			
Urolithins in urine after raspberry drink consumption by human subjects	UPLC	DAD-Q-MS	Same Conditions with Q-TOF	
		Targeted analysis		
		Detection of potential ellagic acid derived metabolites		
		Quantification of urolithins Available authentic standards		
	**Identification/Quantification**			
Urolithins in extracts of mouse plasma, liver, prostate, colon tissue, and luminal content after consumption of black raspberries	UPLC	TQD-ESI^+^-MS/MS	Column: BEH C18 RP (2.1 × 50 mm^2^, 1.7 μm) Eluents: (A) 1.0% HCOOH in H2O; (B) 1.0% HCOOH in AC Gradient: 10% B (0.5 min); 55.5% B (5 min); linear increase to 95% B (6 min); 10% B (9 min) Run (t): 9 min Flow rate: 0.3 mL/min T: 40 °C	[79]
		Tentative Identification Methyl urolithin C and dimethyl ellagic acid were (Accurate mass and fragmentation)		
		UV absorption of urolithins was also consistent with the MS/MS analysis		
	**Identification/Quantification**			
Ellagitannins from blackberry at simulated GI and colonic levels	HPLC	UV-PDA	Column: Phenomenex Gemini (4.6 × 250 mm^2^, 5.0 μm) Eluents: (A) 2.0% acetic acid in H2O; (B) 0.5% acetic acid in 50% aqueous ACN Gradient: 10% B (0 min); 15% B (13 min); 25% B (20 min); 55% B (50 min); 100% B (54 min); 10% B (60 min) Run (t): 60 min Flow rate: 1.0 mL/min Detection Wavelength: 254 and 280 nm T: 30 °C Quantification of ellagitannins: ellagic acid	[81]
	**Identification/Characterization**			
Urolithins and ellagic acid metabolites produced in different animals after consumption of ellagitannins (blackberries)/Biological samples (feces, urine, plasma, urine, ruminal fluid, and feces)	HPLC	DAD	Column: LiChroCART RP C18 (4.0× 250 mm^2^, 5 μm) Guard Column: Same packing. Eluents: (A) 5.0% HCOOH in H2O; (B) ACN Gradient: 1–25% A (0–20 min); 25–55% A (20–30 min); 90% (30–35 min); 90–1% (35–45 min) Run (t): 45 min Flow rate: 1.0 mL/min Detection wavelength: 280, 305, 360 nm	[119]
	**Identification/Characterization**			
	HPLC	DAD-MS/MS	Conditions similar to HPLC-DAD	
	**Identification/Characterization**			
	HPLC	ESI-Q-TOF-MS/MS	Gradient, Column, Run (t): Same as for HPLC-DAD/HPLC-DAD-MS/MS Eluents: (A) H2O: HCOOH in H2O (99.9: 0.1 (*v/v*)); (B) ACN Flow rate: 0.7 mL/min	
	**Identification/Characterization**			
Urine and plasma samples from men consumed blackberry rich products (confection or nectar)	UPLC	TQD-ESI^+^-MS/MS	Column: BEH C18 RP (2.1 × 50 mm^2^, 1.7 μm) Eluents: (A) 1.0% HCOOH in H2O; (B) 1.0% HCOOH in ACN Gradient: 10% B (0.5 min); linear increase to 55.5% B (4 min); 88.9% B (5 min); re-equilibration (6.5 min) Run (t): 9 minFlow rate: 0.75 mL/min T: 40 °C	[129]
		Tentative Identification Q-TOF for methyl urolithin C and DMEA (Accurate mass and fragmentation, UV spectra) Targeted compounds Urolithin A, B, C, D, hydroxyl urolithin C, dimethyl ellagic acid, methyl urolithin A, dimethyl urolithin A		
		Targeted compounds Urolithin A, B, C, D, hydroxyl urolithin C, dimethyl ellagic acid, methyl urolithin A, dimethyl urolithin A		

## 5. Conclusions

Several studies have focused during the last decades on the identification and quantification of (poly)phenol metabolites in *Rubus* genus owing to the high content of its bioactive compounds such as anthocyanins and ellagitannins. The development of analytical technologies, such as hyphenated methods, including HPLC and/or UPLC coupled to high resolution MS, or NMR, have enabled the identification and profiling of a broad range of (poly)phenol metabolites in blackberries and raspberries, even at substantially low concentrations. Nonetheless, further development in analytical capacities could provide a better profiling of complex biological mixtures containing known or yet unknown metabolic products. In parallel, the chemical synthesis or commercial availability of more metabolite standards, could reduce the restrictions associated with the evaluation of (poly)phenol metabolism, particularly when accurate quantitative determination is required. In conclusion, despite the strong indications through the application of advanced analytical techniques, that (poly)phenol metabolites from berry fruits are absorbed to varying degrees and deliver positive effects at various levels in the human body, more in vivo studies should be performed to establish this effect.
